# PROTECTIVE FACTORS IN THE CONTEXT OF SUCCESSFUL AGING IN URBAN-DWELLING ALASKA NATIVE ELDERS

**DOI:** 10.1093/geroni/igz038.1232

**Published:** 2019-11-08

**Authors:** Steffi M Kim, Jordan P Lewis

**Affiliations:** University of Alaska Anchorage, Anchorage, Alaska, United States

## Abstract

Recently, researchers have been exploring successful aging in rural communities of Alaska as it is experienced by Alaska Native Elders. Due to outmigration based on economic, medical, or familial influences, many Alaska Native elders leave their home communities to live in urban settings in Alaska, even though research suggests that most elders would like to remain in their home communities to grow old. Very little is known about the relocation process and how it impacts an elder’s views on successful aging. While established protective factors in rural communities involve family and extended family members, community, subsistence, and traditional activities, there is little knowledge of which protective factors exist in urban settlements supporting successful aging. This exploratory, qualitative study presents the protective factors of successful aging in the context of relocation of Alaska Native Elders from rural to urban dwellings. A life course approach and discourse analysis were used to analyze individual interviews asking how Alaska Native Elders experienced relocation and how successful aging was experienced similarly and differently in rural and in urban settings. A community-based participatory approach allowed for collaboration between researchers and communities as equal partners at all phases of the process, both contributing their expertise to enhance understanding of successful aging and supportive factors. Access to informal supports and meaningful community engagement were more important to rural Elders, and access to health care services and family engagement were important to Elders in urban communities. Challenges remain for Elders in urban environments to establish a sense of community.

